# Habitat Selection Response of Small Pelagic Fish in Different Environments. Two Examples from the Oligotrophic Mediterranean Sea

**DOI:** 10.1371/journal.pone.0101498

**Published:** 2014-07-03

**Authors:** Angelo Bonanno, Marianna Giannoulaki, Marco Barra, Gualtiero Basilone, Athanassios Machias, Simona Genovese, Sergey Goncharov, Sergey Popov, Paola Rumolo, Massimiliano Di Bitetto, Salvatore Aronica, Bernardo Patti, Ignazio Fontana, Giovanni Giacalone, Rosalia Ferreri, Giuseppa Buscaino, Stylianos Somarakis, Maria-Myrto Pyrounaki, Stavroula Tsoukali, Salvatore Mazzola

**Affiliations:** 1 Consiglio Nazionale delle Ricerche, Institute for Coastal and Marine Environment (IAMC), Detached Units of Capo Granitola (TP), Mazara del Vallo (TP) and Naples, Italy; 2 Hellenic Centre for Marine Research, Institute of Marine Biological Resources, Iraklion, Greece; 3 Russian Federal Research Institute of Fisheries and Oceanography (VNIRO), Moscow, Russia; 4 Consiglio Nazionale delle Ricerche (CNR), Roma, Italy; Technical University of Denmark, Denmark

## Abstract

A number of scientific papers in the last few years singled out the influence of environmental conditions on the spatial distribution of fish species, highlighting the need for the fisheries scientific community to investigate, besides biomass estimates, also the habitat selection of commercially important fish species. The Mediterranean Sea, although generally oligotrophic, is characterized by high habitat variability and represents an ideal study area to investigate the adaptive behavior of small pelagics under different environmental conditions. In this study the habitat selection of European anchovy *Engraulis encrasicolus* and European sardine *Sardina pilchardus* is analyzed in two areas of the Mediterranean Sea that largely differentiate in terms of environmental regimes: the Strait of Sicily and the North Aegean Sea. A number of environmental parameters were used to investigate factors influencing anchovy and sardine habitat selection. Acoustic surveys data, collected during the summer period 2002–2010, were used for this purpose. The quotient analysis was used to identify the association between high density values and environmental variables; it was applied to the entire dataset in each area in order to identify similarities or differences in the “mean” spatial behavioral pattern for each species. Principal component analysis was applied to selected environmental variables in order to identify those environmental regimes which drive each of the two ecosystems. The analysis revealed the effect of food availability along with bottom depth selection on the spatial distribution of both species. Furthermore PCA results highlighted that observed selectivity for shallower waters is mainly associated to specific environmental processes that locally increase productivity. The common trends in habitat selection of the two species, as observed in the two regions although they present marked differences in hydrodynamics, seem to be driven by the oligotrophic character of the study areas, highlighting the role of areas where the local environmental regimes meet ‘the ocean triad hypothesis’.

## Introduction

During the last few years the identification of suitable habitat for pelagic fish species represented one of the prominent challenges in fishery research community [e.g. 1–4]. Even though it is widely accepted that the habitat selection by fish species follows the “ideal and free distribution” theory [5], a large number of factors can modulate this tendency, making it difficult to interpret the way fish species select their own “favorable” habitat in different sea areas.

Several studies have been focused recently on small pelagic fish (mainly sardine and anchovy) habitat selection by means of different methodologies: generalized additive modeling [e.g. 1,3,4,6,7], quotient analysis [e.g. 8–11], randomization tests [e.g, 12,13] and geostatistical analysis [e.g. 14]. Environmental preferences are species specific and largely depend on local area conditions. Moreover, even when the same environmental variable are found influential among different areas, the preferred ranges may vary considerably. The Mediterranean Sea is generally considered an oligotrophic area, being at the same time highly heterogeneous in terms of hydrography, bathymetry and productivity. Anchovy (*Engraulis encrasicolus*) and sardine (*Sardina pilchardus*) population dynamics and distribution patterns are known to be strongly dependent on the environment [4], [15], representing an important amount of the total small pelagic fish catches in the Mediterranean [16].

**Figure 1:**
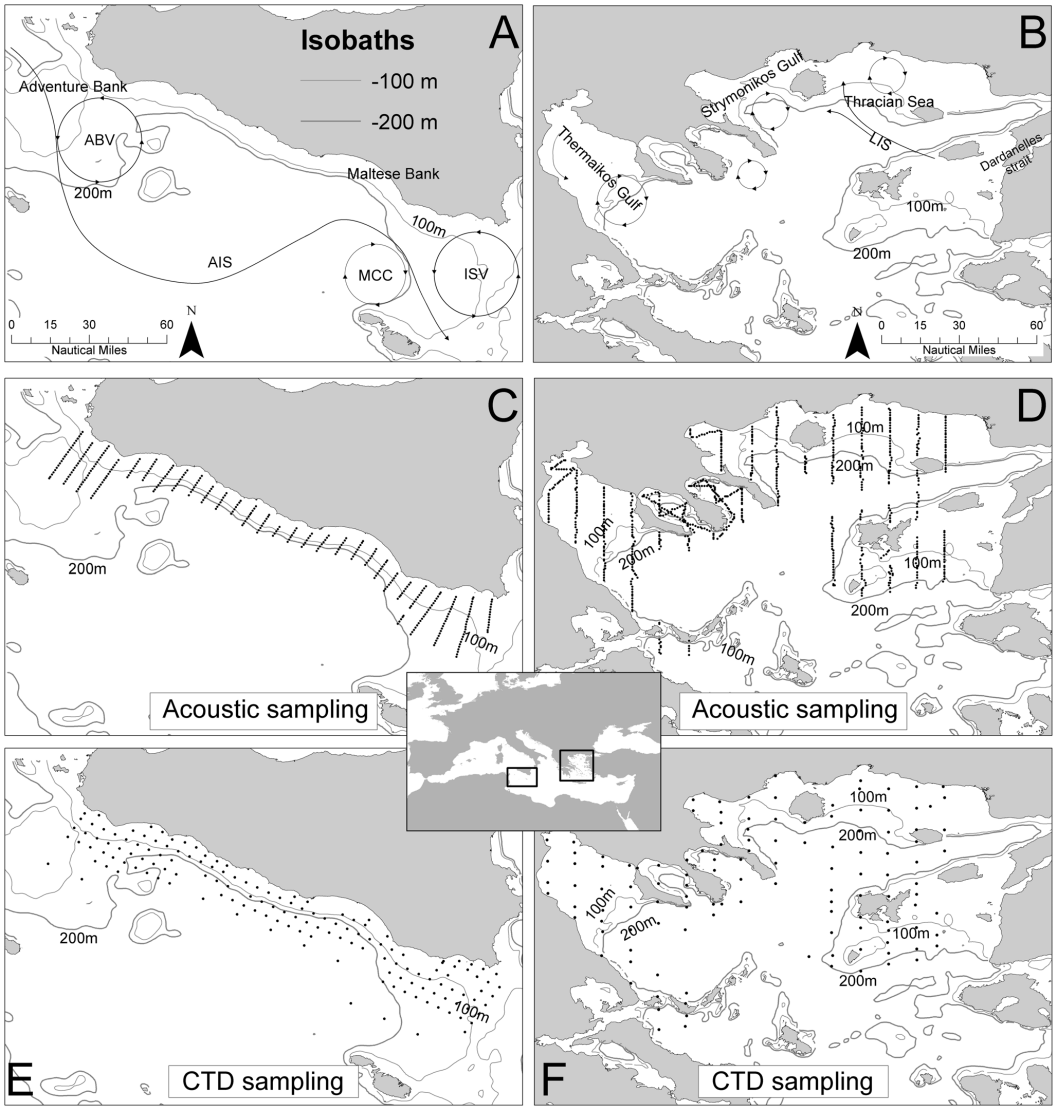
Study areas and sampling design in the Strait of Sicily (A, C, E) and in the North Aegean Sea (B, D, F).

In the current work, a large environmental dataset, composed by *in situ* measurements and satellite data, is used to study and highlight differences in the habitat selection behavior of anchovy and sardine in two areas: the Strait of Sicily and the North Aegean Sea. The Strait of Sicily (Fig. 1a) is characterized by a complex circulation, since it is the area connecting the two main basins of the Mediterranean Sea. The Modified Atlantic Water (MAW), fresher and warmer, flows in the upper layer towards the eastern Mediterranean basin whereas the Levantine Intermediate Water (LIW), saltier and colder, moves in the opposite direction [17], [18]. The general surface circulation pattern is locally controlled by the motion of the MAW, which bifurcates in the Atlantic Ionian Stream (AIS), a meandering surface current flowing towards the Ionian Sea, and the Atlantic Tunisian Current (ATC) flowing southward [17], [19]. During summer, the Atlantic water advected by the AIS to the south of Sicily is warmer than its north surrounding waters at the same depth. The AIS motion produces a cyclonic vortex over the Adventure Bank (Adventure Bank Vortex – ABV) and an almost permanent upwelling along the southern shore of the island [18]. The North Aegean Sea (Fig. 1b) is characterized by high hydrological complexity mostly related to the Black Sea water (BSW) which enters the Aegean Sea through the Dardanelles Strait. The overall circulation is mainly determined by the presence of the Limnos-Imvros stream (LIS), which carries waters of Black Sea origin onto the Samothraki plateau [20], generating a permanent anticyclonic system. The outflow of BSW (salinity <30) enhances local productivity, and its advection in the North Aegean Sea induces high hydrological and biological complexity [21], [22]. This is further enhanced by the presence of a series of large rivers flowing out into semi-closed basins like Thermaikos Gulf, enhancing the productivity locally.

In these areas, acoustic data acquisition for small pelagics has been conducted since 1998 [23] and 1995 [6], respectively. Surveys took place in both areas during summer, which corresponds to the peak of the spawning period for anchovy [10], [11], [24], [25] and the recruitment period for sardine [26]. Previous studies in the basin have focused on habitat suitability modeling for these species, using presence/absence information and determining the areas with suitable conditions to support anchovy and sardine presence [1], [4], [27]. In this work we analyze the environmental preference and processes driving the habitat selection of anchovy and sardine populations by taking into account the spatial distribution of species density in relation to environmental patterns. Specifically, we focus on high density values, seeking for similarities or differences in the “mean” behavioral patterns for each species. Two kinds of variables were selected for this study: one group of factors related to or being proxies of the trophic status of the water column (e.g. chlorophyll and particulate organic carbon) and a group of variables which describe the hydrographic regimes in each area (e.g. temperature, salinity, potential energy deficit and kinetic energy).

## Materials and Methods

The study was carried out on data collected in two sea areas without any restriction. We received the necessary permissions for working at sea in National waters by the Italian and Greek Coastal Guards for the Strait of Sicily and the North Aegean Sea respectively.

The study areas are:

in the Strait of Sicily between 35°N and 38°N latitudes and between 11°E and 16°E longitudes;
in the North Aegean Sea between 38°N and 41°N latitudes and between 22°E and 27°E longitudes.

We state that no specific permissions were required for the surveyed area both in the Strait of Sicily and in the North Aegean Sea, since the involved Institutes (IAMC-CNR and HCMR) were encharged by their Ministries to carry out the data collection in the framework of European Data Collection Framework (DCF - Reg.Ce. N° 199/2008, N° 665/2008 and Commission Decision N° 949/2008).

We confirm that the field studies did not involve endangered or protected species.

Biological samples (anchovies and sardines) were collected by means of a pelagic trawl net during the surveys at sea, with the main aim of evaluating species composition and size classes distribution. Due to the low resistance of small pelagic fishes to catch with net, the specimens used for the study were already dead when coming on board within the trawling net.

### 1. Acoustic data collection

Acoustic sampling was performed by means of scientific split-beam echosounders working at 38 kHz and calibrated following standard techniques (Foote et al., 1987). Acoustic data were recorded at a speed of 8–10 nmi h^−1^. Minimum sampling depth varied between 10 to 20 m depending on the area. The size of the Elementary Distance Sampling Unit (EDSU) was one nautical mile (nmi, 1.852 km). We considered as anchovy/sardine presence any school or echo assigned to anchovy/sardine either by echo trace classification or attributed to anchovy/sardine based on the catch composition of identification hauls [28]. Midwater pelagic trawl sampling was carried out in order to identify and verify anchovy and sardine echo traces and length frequency.

Acoustic data analysis was performed using the Myriax Echoview software. Anchovy and sardine density (t/nmi^2^) for each EDSU was evaluated by merging the biological and acoustic data, based on the nearest haul method [28].

The study area in the Strait of Sicily comprises the continental shelf along the southern coast of Sicily (Fig. 1c). The echosurvey sampling strategy adopted parallel transects characterized by 5 nmi inter-transect distance (Fig. 1c and Table 1). The investigated bathymetric range was 20–300 m.

**Table 1:** Details on the echosurveys carried out in the Aegean Sea and Strait of Sicily.

Period of the Echosurvey	Area	Research vessel	Echosounder	CV_Geo_
2003 July	Aegean Sea	Philia	Biosonic DT-X	0.27
2004 June	Aegean Sea	Philia	Biosonic DT-X	0.32
2005 June	Aegean Sea	Philia	Biosonic DT-X	0.23
2006 June	Aegean Sea	Philia	Biosonic DT-X	0.26
2008 June	Aegean Sea	Philia	Biosonic DT-X	0.21
2002 July	Strait of Sicily	G. Dallaporta	Simrad EK500	0.16
2003 June	Strait of Sicily	G. Dallaporta	Simrad EK500	0.11
2005 June	Strait of Sicily	G. Dallaporta	Simrad EK60	0.10
2006 June	Strait of Sicily	G. Dallaporta	Simrad EK60	0.13
2007 July	Strait of Sicily	G. Dallaporta	Simrad EK60	0.15
2008 August	Strait of Sicily	G. Dallaporta	Simrad EK60	0.13
2009 July	Strait of Sicily	G. Dallaporta	Simrad EK60	0.11
2010 July	Strait of Sicily	G. Dallaporta	Simrad EK60	0.15

CV_Geo_ is the coefficient of variation related tothe survey precision and the spatial sampling error in the estimate of the high density areas, estimated by means of geostatistics (Rivoirard et al., 2000).

In the North Aegean Sea acoustic surveys were carried out along predetermined parallel transects with 10 nmi inter-transect distance in open areas, whereas zigzagged transects were sampled inside gulfs (Fig. 1d and Table 1).

### 2. Environmental dataset

Satellite and *in situ* measurements were used in this study to infer the environmental conditions in the study areas and to investigate the habitat suitability of the considered species. Satellite dataset are largely used in fishery community due to their consistent space-time coverage and their ability to highlight specific ocean processes. Furthermore, ETOPO1 bathymetric data were used to associate bottom depth values at each observation. The ETOPO1 dataset, provided by NOAA (http://www.ngdc.noaa.gov/mgg/global/global.html), is built by merging a number of global and regional datasets, integrating land topography and ocean bathymetry over a 1 arc-minute grid.

#### 2.1 In situ measurements

In the Strait of Sicily water column parameters were collected during co-occurring hydrological surveys carried out on board the R/V “Urania” covering the same area as the echosurveys. The sampling strategy (Fig. 1e) was based on a regular grid with 4 nmi mesh size in the coastal sector and 12 nmi mesh size in offshore waters. Salinity, temperature and pressure profiles, collected from surface down to bottom or 600 m (maximum depth) by means of SBE911 plus CTD probe (Sea-Bird Inc.), were processed using Seasoft-Win32 software following the Mediterranean and Ocean Data Base instructions [29]. Density (σ_θ_) was also derived from the aforementioned parameters. In the North Aegean Sea hydrographic sampling was performed over a grid of 205 CTD stations sampled concurrently with acoustics on board the R/V “Philia”. The sampling strategy (Fig. 1f) was based on a coarser regular grid (10 nmi mesh size). At each station vertical profiles of temperature and salinity were obtained from surface down to bottom or 200 m (maximum depth) using a SBE-25Seacat internally recording CTD unit (Sea Bird Electronics) and processed using Seasoft-Win32 software.

Since anchovy and sardine biomass values at each EDSU represented an integrated measure of abundance (t/nmi^2^) along the water column, we simplified the CTD vertical profiles breaking the water column in two strata, computing for each one the average temperature, salinity and density. Stratification followed the approach proposed by Laprise and Pepin [30], where the Upper Mixed Layer (UML) is defined as the layer from the surface down to the depth where temperature is 1°C higher than that of bottom waters, and the Bottom Layer (BL) is defined as the layer from the end of the UML down to the bottom or up to 200 m, wherever bottom depth is deeper than 200 m. The breakdown of the water column into UML and BL provided a good description of the stratification of the water column as well as a rough indication of the water circulation.

Along with mean temperature, salinity and density, also the UML thickness and the Potential Energy Deficit (PED) [7] were considered in our analysis. The PED parameter is a stratification index and can be considered as the energy required to cause dis-stratification of the water column. Specifically, it was calculated using the following equation:
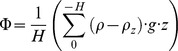
where H is the bottom depth or 200 m in deeper stations, *ρ* is the average density, z is the depth, *ρ_z_* is density at depth z and *g* is the gravity.

The acoustic along-track EDSU was 1 nmi while CTD station were collected for both areas at a coarser grid. Thus UML and BL parameters as well as PED were interpolated by means of bilinear spline interpolation applied in GRASS GIS software [31] using 1 nmi interpolation grid in order to obtain a full link between acoustic and environmental datasets.

#### 2.2 Satellite dataset

In addition to the *in situ* measurements, annual mean monthly values of satellite variables [1], [32] for the study areas and periods were used. Specifically, we considered the Sea Surface Chlorophyll-a (Chl_sat_), the Particulate Organic Carbon (POC), the Chromotophoric Dissolved Organic Matter (CDOM index), the Photosynthetically Available Radiation (PAR), the Kinetic Energy (KE), and the Mediterranean Absolute Dynamic Topography (MADT), which are described in Table 2. The altimeter products are produced by Ssalto/Duacs and distributed by Aviso, with the support of Cnes (http://www.aviso.oceanobs.com/duacs/). Since the spatial resolution of the aforementioned dataset was lower with respect to the acoustic EDSU, each dataset was resampled using 1 nmi grid spacing by means of bilinear spline interpolation.

**Table 2:** Satellite variables description.

Variable	Abbreviation	Sensor/Model	Resolution	Units		Source
Sea SurfaceChlorophyll-a	Chl_sat_	MODIS Aqua	4 km	mg m^−3^	Chlorophyll aconcentration	http://oceancolor.gsfc.nasa.gov
PhotosyntheticallyAvailable Radiation	PAR	MODIS Aqua	4 km	Einsteins m^−2^ day^−1^	Daily integrated photosyntheticallyavailable radiation from 400 - 700 nm	http://oceancolor.gsfc.nasa.gov
Particulate OrganicCarbon	POC	MODIS Aqua	4 km	mg m^−3^	Particulate organiccarbon concentration	http://oceancolor.gsfc.nasa.gov
Chromophoric DissolvedOrganic Matter	CDOM Index	MODIS Aqua	4 km	Dimensionless	Chlorophyll-chromophoricdissolved organic matterproportion index	http://oceancolor.gsfc.nasa.gov
KineticEnergy	KE	AVISO	∼14 km	cm^2^ s^−2^	Calculated as the product of0.5*(U^2^+V^2^) where U: longitudinal geostrophic velocity and V: latitudinal geostrophicvelocity	http://aviso.oceanobs.com/
Mediterranean AbsoluteDynamic Topography	MADT	AVISO	∼14 km	cm	Sea surface heightwith respect to the geoid	http://www.aviso.oceanobs.com/en/data/products/sea-surface-height-products/regional/madt-mediterranean-sea.html

### 3. Statistical methods

#### 3.1 Habitat selection

Anchovy and sardine selection behavior for the abovementioned environmental variables was evaluated considering the two study areas in the period 2002–2010 for the Strait of Sicily and 2003–2008 for the North Aegean Sea. The Single Parameter Quotient analysis [8], [9], [22], [33], [34] was used to investigate the “mean” spatial behavior of species in the specific temporal windows. To this aim, QI analysis was performed on two datasets, one for each study area, composed by all data (per EDSU) pooled.

The first step in applying quotient analysis was the identification of the specific class intervals for each environmental variable. We ensured that the minimum occurrence per category was not less than 5% and the maximum one did not exceed 25% of all measurements. In addition the range in each interval was chosen in order to reflect the regional variability [*sensu* 22,34]. Thus, for each interval the Quotient index (QI) was obtained through the following formula:
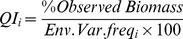
where *i* represents the *i-th* frequency histogram interval.

To test the significance of the observed QI values, randomization procedure was used [33] to calculate the confidence intervals (CI – dashed lines in QI plots) for the null hypothesis (i.e. random association between biological and environmental variable). Avoidance or selection were subsequently evaluated on the basis of the calculated CI. In particular, significant selection is evidenced when QI values are higher than or equal to the upper CI, while significant avoidance corresponds to QI values lying below or equal to the lower CI. QI values between the two CI curves are interpreted as tolerance behavior [33].

**Figure 2:**
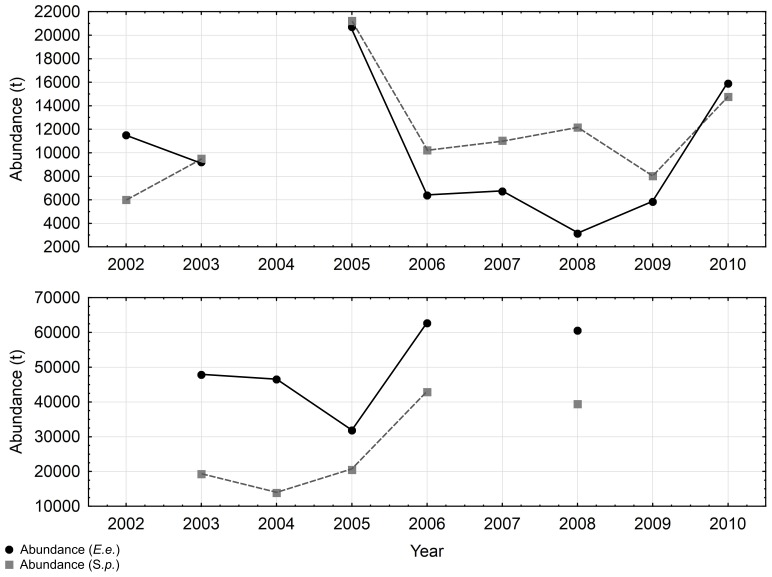
Biomass estimates of *Engraulis encrasicolus* (E.E.) and *Sardina pilchardus* (S.P.) in the Strait of Sicily (upper part) and in the North Aegean Sea (lower part).

Since the biomass of both species showed high inter-annual variability in both areas (Fig. 2), higher biomasses are expected to influence the results of QI analysis. To reduce such bias, standardization of density values among years was adopted. Specifically, for each year and study area, the average density was computed; then the density values per EDSU and survey were divided by the average density value corresponding to the same survey.

#### 3.2 Analysis of ecosystem differences in terms of habitat selection

Principal Component Analysis (PCA) was applied in each study area separately, using all environmental variables. It is a data reduction technique and is often used to identify common pattern within a large dataset. Zwolinski et al. [35], analyzing the sardine potential habitat along the western Portuguese continental shelf, used PCA to infer the presence of structures in environmental data and considered the identified pattern as main effect (interaction term) in GAM models, highlighting the relationships between the structured variability of environmental dataset and sardine distribution. Similarly, in the present work the relationship between the environmental patterns, identified by means of PCA analysis, and fish density was assessed using the PCA factor coordinates (i.e. the observation values on each PCA axis obtained after the system was rotated and centered) as “environmental variable” in the QI analysis. In this way a sort of multivariate application of QI analysis was obtained, which permitted to relate the identified physical processes and habitat selection. Principal components were computed via the correlation matrix and, in interpreting identified patterns, only variables presenting a correlation with the PC axis higher than 0.5 were considered.

## Results

The analysis of the environmental dataset permitted to highlight the main environmental differences between the two areas. Specifically, significant difference (Mann-Whitney U Test, p<0.001) in the UML temperature (Table 3) was evidenced between the two areas, despite the magnitude of such difference was not so high (slightly higher UML temperature values in the North Aegean Sea than in the Strait of Sicily). On the contrary, median UML salinity presented a narrower range of values in the Strait of Sicily compared to the North Aegean Sea, being lower (Mann-Whitney U Test was significant at p<0.001) at the second area; this difference is largely due to the influence of the fresher BSW in the upper layer. In both areas the thickness of UML showed similar values (i.e. not significant difference) with a median value in the range 24.0 m - 37.0 m (Table 3). Moreover, median temperature in the BL assumes slightly higher values (p<0.001) in the Strait of Sicily than in the North Aegean Sea, while significantly lower salinity values (p<0.001) were recorded in the former area. Such environmental conditions in the BL are mainly driven by the MAW flowing on the southern continental shelf south of Sicily. In order to avoid possible interaction with bottom depth, a correlation analysis between bathymetry and all environmental variables was carried out in each area. Particularly, BL density and BL thickness showed significant positive correlation with bathymetry in both areas (Table 4), thus they have not been considered in the following analyses.

**Table 3:** Median values of the *in situ* and satellite variables in the two study areas.

	Strait of Sicily								North Aegean Sea				
	2002	2003	2005	2006	2007	2008	2009	2010	2003	2004	2005	2006	2008
UML Salinity (PSU)	37.7	37.9	37.7	37.7	37.5	37.5	37.6	37.6	36.2	36.3	36.7	35.8	37.2
UML Temperature (°C)	19.1	19.7	18.8	18.7	19.2	19.7	18.8	18.4	20.0	18.5	18.6	19.3	20.4
UML Thickness (m)	31.0	32.0	27.0	32.0	30.0	30.0	24.0	36.0	25.0	35.0	32.0	24.0	37.0
BL Temperature (°C)	15.5	15.2	14.7	14.9	15.8	15.6	15.0	15.2	13.8	14.0	14.3	14.1	15.0
BL Salinity (PSU)	38.2	38.4	38.0	38.1	38.0	38.0	38.1	38.1	38.5	38.5	38.6	38.4	38.7
PED (kg m^−2^ s^−2^)	159.7	160.2	147.2	138.7	152.4	152.6	122	139	353.9	291.5	274.2	319.9	317.6
Chl_sat_ (mgm^−3^)	0.14	0.13	0.16	0.10	0.14	0.13	0.12	0.12	0.25	0.25	0.22	0.30	0.22
CDOM	4.7	5.7	4.8	3.8	4.4	4.5	5.7	5.8	7.5	5.5	5.7	6.6	6.5
POC (mgm^−3^)	49.0	46.0	53.0	39.0	48.0	46.0	43.0	44.0	72.6	69.8	66.2	85.3	66.2
KE (cm^−2^s^−2^)	453.0	126.0	101.0	71.0	597.0	297.5	157.0	135.5	60.6	40.9	43.0	20.8	37.8
PAR (Einsteinsm^−2^ day^−1^)	58.0	59.0	59.0	57.0	59.0	59.0	60.0	59.0	59.4	54.8	57.3	56.0	57.6
MADT (cm)	−0.6	−0.6	0.6	4.3	5.8	3.8	3.4	2.7	6.7	6.1	9.1	7.1	7.7

**Table 4:** Pearson correlation coefficient between bathymetry in both study areas and the considered environmental variables.

	North Aegean Sea	Strait of Sicily
UML Temperature	ns	ns
UML Salinity	ns	ns
UML Thickness	ns	ns
BL Temperature	ns	−0.43
BL Salinity	ns	0.56
BL Density	0.48	0.62
BL Thickness	0.68	0.80
CDOM	ns	ns
Chl_sat_	ns	−0.41
PAR	ns	ns
POC	Ns	−0.42
KE	ns	ns
MADT	ns	ns
PED	ns	0.52

Only significant values (p<0.001) are reported. ns: non significant.

Among satellite variables Chl_sat_, CDOM index and POC showed significantly higher (p<0.001) values in the Northern Aegean Sea than in the Strait of Sicily, confirming the lower productivity of the Strait of Sicily singled out in other studies [32], [36]. Significantly higher values of KE (p<0.001) in the Strait of Sicily are due to the particular position of this area that is characterized by high dynamics of water masses. A more stable stratification of the water column in the North Aegean Sea is evidenced by the significantly higher (p<0.001) PED values in this area (Table 3). Most part of the surveyed area in the Strait of Sicily is located within the coastal waters, characterized by low MADT values. This circulation feature is associated with upwelling processes, whereas higher values observed in North Aegean Sea are associated with the presence of anti-cyclonic vortexes related to BSW influence.

**Figure 3:**
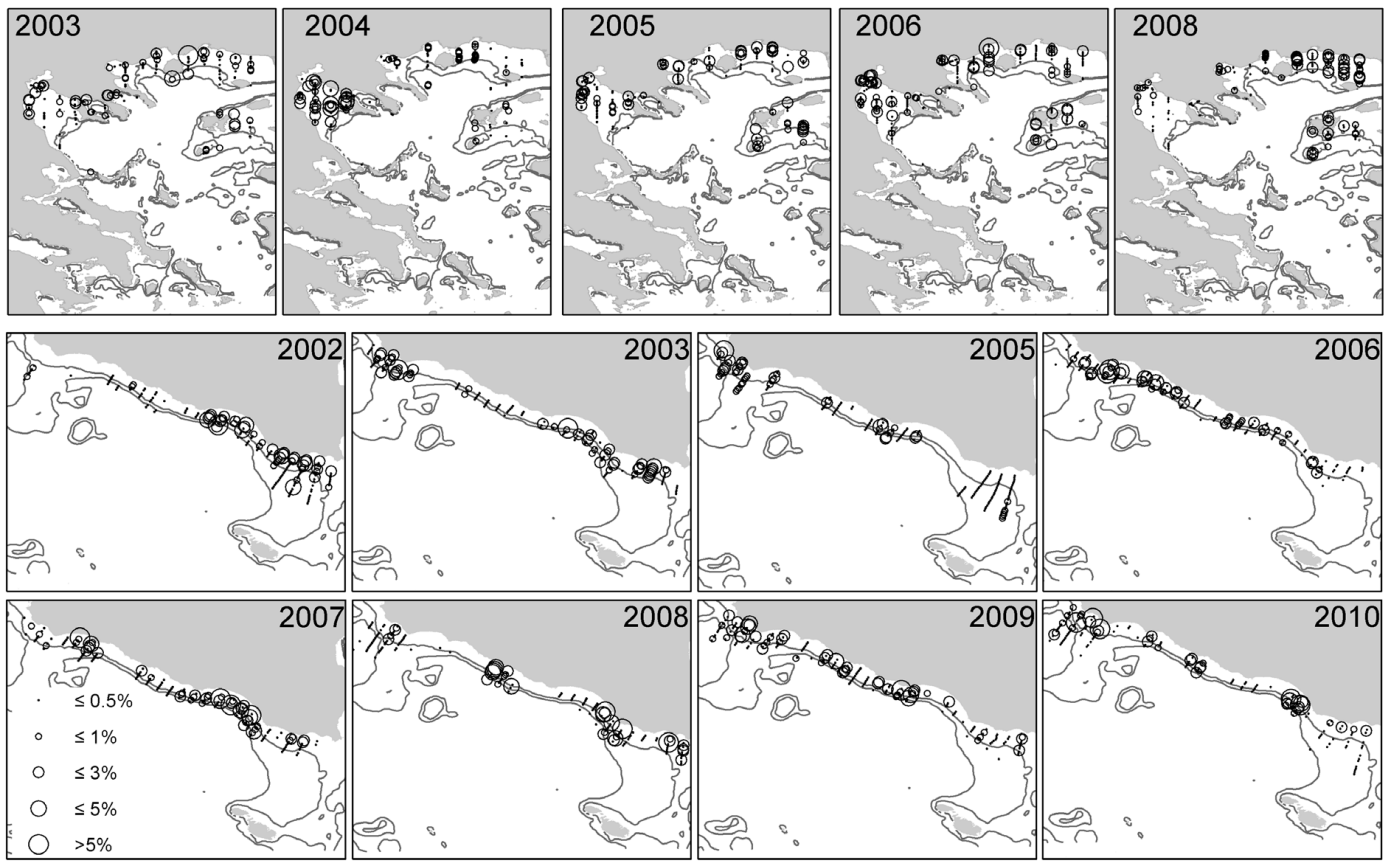
Anchovy distribution maps in the North Aegean Sea (upper part) and in the Strait of Sicily (lower part). Symbols size is proportional to the abundance of strictly positive values, while zero values are omitted.

**Figure 4:**
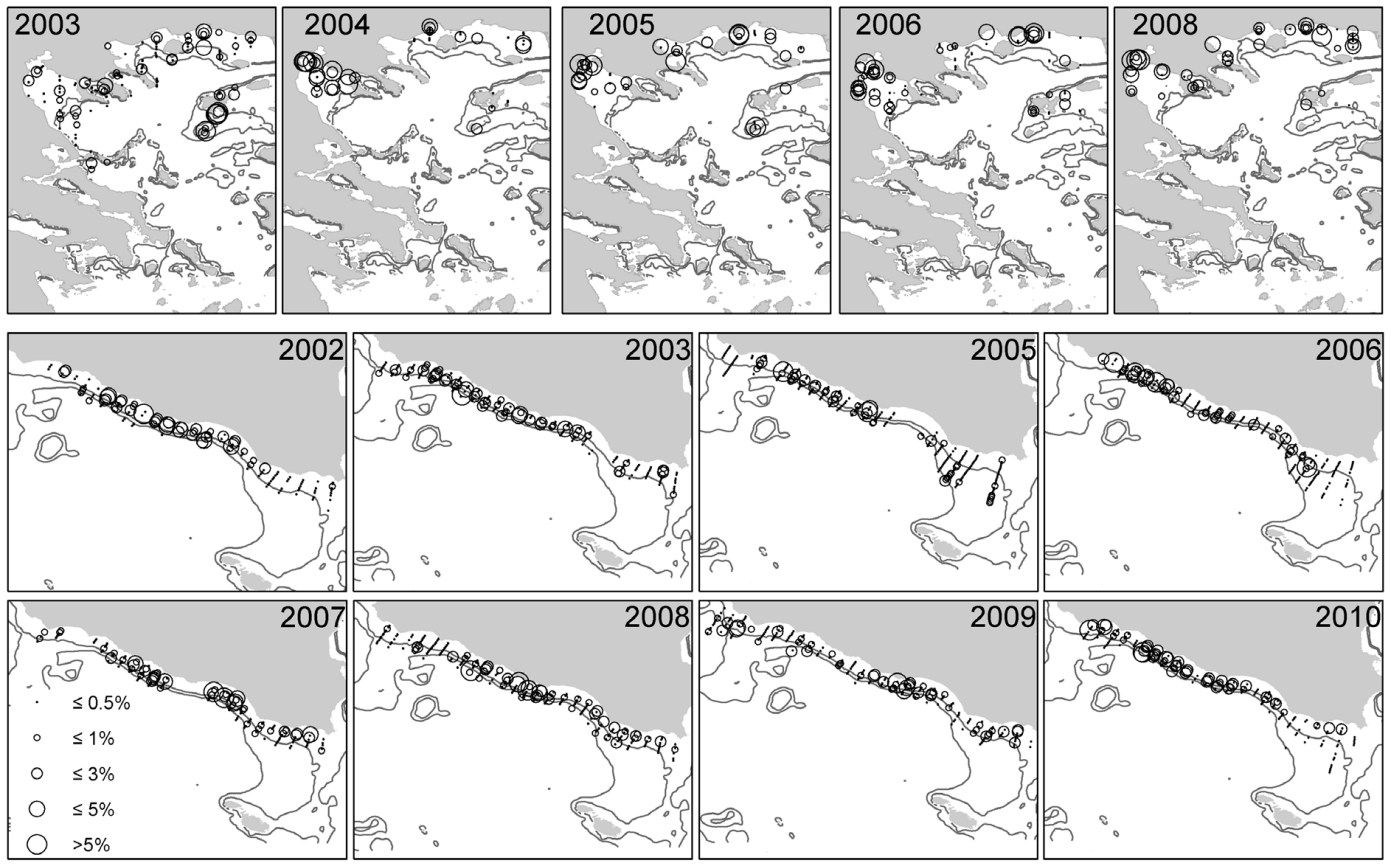
Sardine distribution maps in the North Aegean Sea (upper part) and in the Strait of Sicily (lower part). Symbols size is proportional to the abundance of strictly positive values, while zero values are omitted.

During the study period both anchovy and sardine showed higher biomasses in the North Aegean Sea than in the Strait of Sicily (Fig. 2). The central part of this latter area (Figs. 3 and 4) is occupied by both species consistently in each survey, while their presence in the Maltese and Adventure banks shows high variability. Furthermore, anchovy shows a more patchy behavior than sardine, mainly characterized by higher biomass values over the Adventure bank than on the Maltese bank, except in 2002 and 2008 (Fig. 3). The opposite happens for sardine, showing higher biomass values in the Maltese Bank (except in 2009) than in the Adventure Bank (Fig. 4). In the Northern Aegean Sea both species present higher densities over the coastal areas and the semi-closed areas receiving river outflows (Figs. 3 and 4). Opposed to the Strait of Sicily, anchovy generally presents less patchy, more uniform distribution compared to sardine. In 2003 and 2006, years with the highest sardine biomass, the species shows a more uniform distribution and ubiquitous behavior compared to other years. In both study areas the analysis of the haul catches from the acoustic surveys showed the dominance of adults in anchovy population (ages 1 and 2) and the dominance of both juveniles and adults in sardine population (ages 0, 1 and 2) [37].

**Figure 5:**
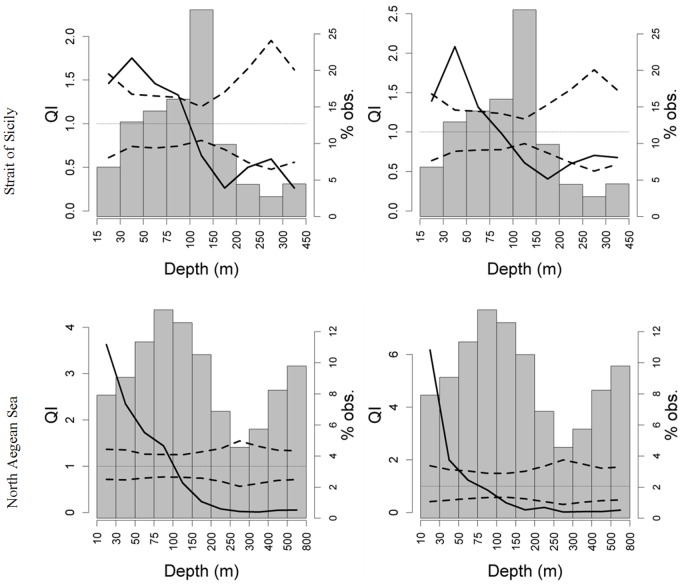
QI plots related to depth for *Engraulis encrasicolus* (left) and *Sardina pilchardus* (right) in both study areas. Each plot shows the observed QI curve (solid line), its CI (dashed lines), and the frequency histogram of depth. The dotted line in each plot indicates the value QI = 1.

**Figure 6:**
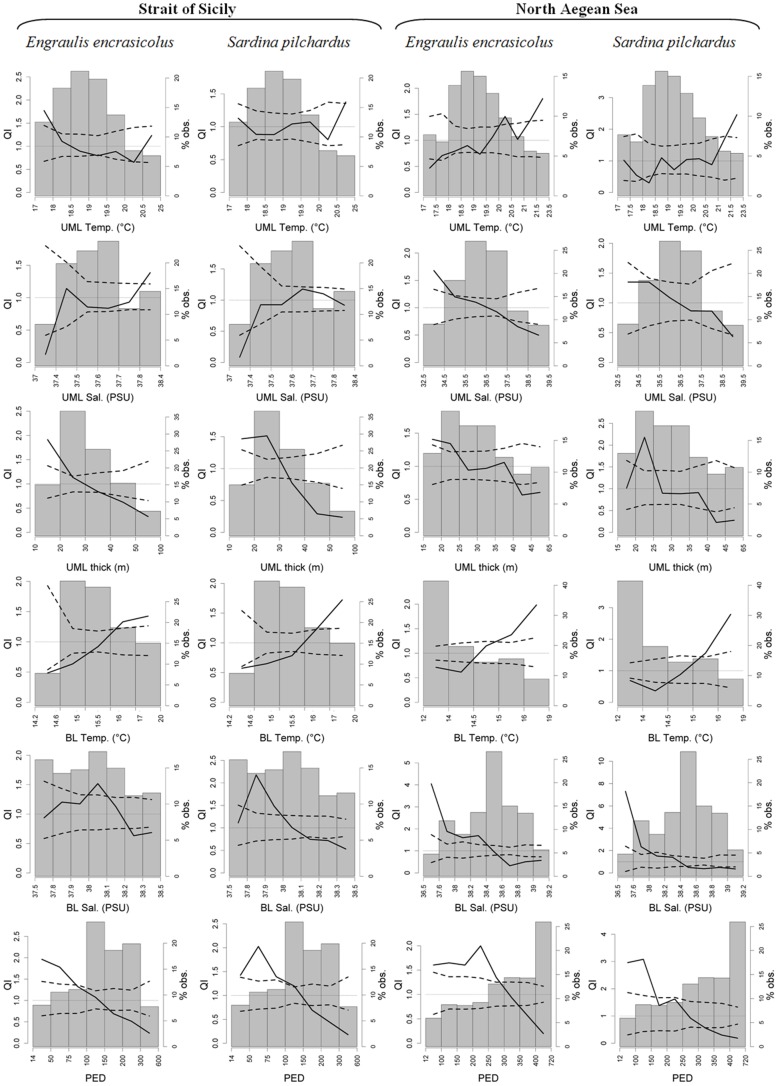
Quotient curves related to water column parameters for *Engraulis encrasicolus* and *Sardina pilchardus* in both study areas. Each plot shows the observed QI curve (solid line), its CI (dashed lines), and the frequency histogram of the considered environmental variable. The dotted line in each plot indicates the value QI = 1.

The quotient curves relative to bottom depth (Fig. 5) indicate the anchovy and sardine selection for shallower waters and a marked avoidance behavior for deeper waters in both areas. A different bathymetric selection, although less pronounced between anchovy and sardine is evident: in the Strait of Sicily anchovy shows a selection in the bathymetric range of 30–100 m, while for sardine the depth selection range is 30–75 m. A similar result is also shown in the North Aegean Sea where the selected bathymetric range for anchovy is 10–100 m, while sardine shows selective behavior for a narrower range (10–50 m). In addition, UML temperatures higher than 21°C are selected by anchovy and sardine in North Aegean Sea, where anchovy also selects lower UML and BL salinity values (<34.5 and <38.4 respectively), and avoids higher ones (>37.5 and >38.6 respectively). Conversely, in the same area sardine demonstrates different selection/tolerance/avoidance behavior in relation to UML and BL salinity with respect to anchovy. Indeed, regarding the UML salinity, a wide tolerance behavior is evidenced by sardine, except for a significant avoidance of salinity values higher than 38.5. In terms of BL salinity sardine selects values lower than 38 avoiding the ones higher than 38.4 (Fig. 6). In the Strait of Sicily higher UML temperatures (>20.5°C) are selected by sardine, while lower temperature values (<18°C) are selected by anchovy, evidencing also a different behavior between the two study areas (Fig. 6). As regards UML salinity, in Sicilian waters both species seem to avoid lower salinity values (<37.4), evidencing more behavioral differences from North Aegean Sea. Furthermore, in Sicily waters anchovy significantly selects UML salinities higher than 37.8, while sardine shows a tolerance behavior for values higher than 37.4. With respect to BL salinity the situation is less clear in the Strait of Sicily where both species visibly selects a very narrow BL salinity range (Fig. 6).

Similar behavior is evidenced in terms of the UML thickness, BL temperatures and PED by both species in the two study areas, despite selection/avoidance ranges slightly varies (Fig. 6). Particularly, in both areas lower UML thickness values are selected and higher ones are avoided, as well as higher BL temperatures are selected a lower avoided. Concerning the PED parameter, all cases show a preference for lower values, reflecting the selection of less stratified waters, as well as an active (i.e. significant) avoidance of high stratified ones. Notably sardine in both areas shows similar selection behavior, selecting PED values lower than 150 kg m^−2^s^−2^.

**Figure 7:**
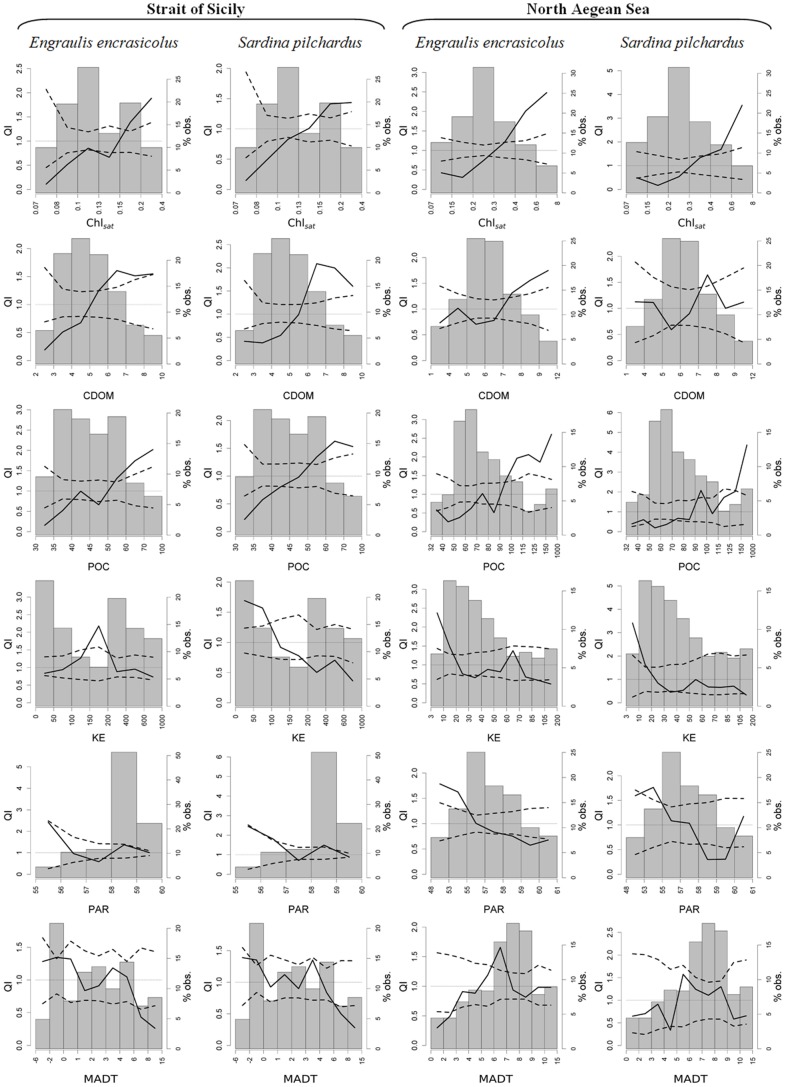
Quotient curves related to satellite data for *Engraulis encrasicolus* and *Sardina pilchardus* in both study areas. Each plot shows the observed QI curve (solid line), its CI (dashed lines), and the frequency histogram of the considered environmental variable. The dotted line in each plot indicates the value QI = 1.

The analysis of quotient curves obtained for satellite parameters (Fig. 7) highlights similar behavior for both species concerning the CDOM index, POC and Chl_sat_ in both areas, despite selection/tolerance/avoidance ranges varies according to the environmental differences characterizing the two study areas. Anchovy and sardine select higher CDOM index, POC and Chl_sat_ values and avoid lower ones (Fig. 7). Quotient curves for KE (Fig. 7) highlight in the North Aegean Sea a clear selective behavior for lower KE values by both species, indicating a preference for less intense water movements. In the Strait of Sicily, mainly due to the occurrence of high water masses dynamics, sardine clearly shows an avoidance behavior for high KE values, while anchovy seems to select an intermediate range of KE (130–210 cm^2^/s^2^). As regards PAR, in the Strait of Sicily both species do not show any clear selection behavior. In North Aegean Sea waters low PAR values (<55 Einsteins m^−2^ day^−1^) are actively selected, while higher PAR values are avoided.

The selectivity curves related to MADT clearly show a different situation between the two areas. In the Strait of Sicily both species avoid MADT values higher than 6 cm, while a selection is shown for waters presenting MADT between −2 cm and 0 cm. In the North Aegean Sea sardine does not seem to be influenced by this parameter, whereas anchovy avoids lower values and selects the ones in the range 6–7 cm.

In order to highlight the presence of structure in the environmental dataset, PCA analysis has been used. PCA results show that in both study areas the first two factors account approximately for 50% of the total variability and can be related to specific processes. Particularly, in the Strait of Sicily (Table 5a) lower values on the 1^th^ PC (hereafter PC1), explaining 37% of the variance, correspond to lower PED, UML thickness, bathymetry and MADT as well as to higher CDOM, CHL_sat_ and POC, and could be linked to areas influenced by weak coastal upwelling. Conversely, the second PCA axis (PC2), accounting for an additional 15% of the variance, is mostly related to water masses dynamics and specifically to variables like KE, salinity (both UML and BL) and MADT. Differently from Sicily waters, in North Aegean Sea PCA analysis (Table 5b) highlights two different processes driving food availability. Higher values on the first PCA axis (explaining 29% of the variance) coincide with higher food availability (higher CDOM, CHL_sat_ and POC), UML temperature and MADT, as well as lower column water salinity and UML thickness, and could be related to the presence of BSW. Furthermore, lower values on the second PCA axis (explaining 19% of the variance) are linked to higher CHL_sat_, POC and BL temperature as well as to lower PED and depth, thus identifying coastal enriched areas that could be affected by the formation of a weak coastal upwelling.

**Table 5:** Factor variables correlation relative to PCA carried out on the environmental variables most influencing anchovy and sardine population.

Area	Variables	PC1	PC2	Area	Variables	PC1	PC2
	CDOM	−**0.76**	0.04		CDOM	**0.59**	0.15
	Chl_sat_	−**0.83**	−0.06		Chl_sat_	0.46	−**0.68**
	PAR	0.16	−0.03		PAR	−0.43	0.09
	POC	−**0.84**	−0.07		POC	**0.52**	−**0.67**
	KE	0.32	−**0.50**		KE	−0.22	0.19
	Depth	**0.61**	0.48		Depth	−0.11	**0.62**
Strait	PED	**0.90**	0.07	North	PED	0.44	**0.71**
of Sicily	UML temp.	−0.01	−0.22	Aegean Sea	UML temp.	**0.56**	−0.02
	UML sal.	−0.39	**0.63**		UML sal.	−**0.85**	−0.21
	UML thick.	**0.77**	−0.14		UML thick.	−**0.74**	−0.09
	BL temp.	−**0.50**	−0.29		BL temp.	−0.32	−**0.62**
	BL sal.	0.42	**0.77**		BL sal.	−**0.79**	0.25
	MADT	**0.61**	−**0.63**		MADT	**0.49**	0.34
	% variance	37	15.7		% variance	29.3	19.05

The analysis was performed separately on each area in order to maximize the comprehension of environmental process affecting each area. Values in bold represent the variables most related to each PCA axis.

**Figure 8:**
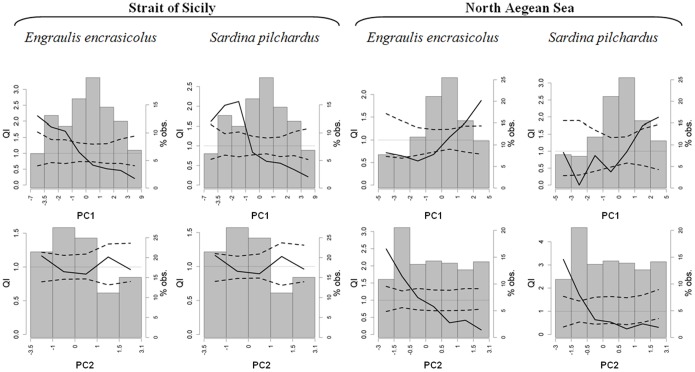
QI plot related to PCA factor coordinates. Each plot shows the observed QI curve (solid line), its CI (dashed lines), and the frequency histogram of the considered environmental variable. The dotted line in each plot indicates the value QI = 1. For a correct interpretation factor variables correlation relative to PCA (Tab. 5) must be considered.

In a further step, and in order to demonstrate the association between identified processes and habitat suitability of anchovy and sardine, a QI analysis (Fig. 8) has been performed on the PCA factor coordinates (i.e. the observation values on each PCA axis obtained after the system was rotated and centered). The results clearly show that habitat suitability is significantly related to identified processes (Fig. 8), except for the one related to the second PCA axis in the strait of Sicily describing a pure physical process not connected to food availability. Indeed in this area, both species show significant selection behavior for lower values of PC1 axis (related to higher food availability), while the QI curve related to the PC2 (the one not related to food availability) lies completely between the two CI (neither selection nor avoidance). In the North Aegean Sea, where higher PC1 values represent high food availability linked to less salty and almost well stratified waters of Black Sea origin (then not linked to depth values), both species clearly select higher values of such axis. Furthermore, in this area the PC2 shows the presence of a secondary effect responsible of food availability, and then anchovy and sardine select lower values of such axis (representing areas characterized by high CHL_sat_ and POC values, lower depth and less stratified waters) actively avoiding higher ones. This is likely to reflect selection behavior driven by the effect of rivers and the occurrence of a weak coastal upwelling.

## Discussion

Anchovies and sardines are known to distribute in various ecosystems within the temperate zone that largely differentiate in terms of oceanographic characteristics and productivity, i.e. highly productive areas such as the California Current, the Humboldt Current, the South African waters, the western Pacific Ocean, the Australian waters and the Northeast Atlantic up to the North Sea. Moreover, they distribute in closed basins like the Black Sea and the Mediterranean Sea. The latter is highly heterogeneous in terms of hydrography, bathymetry and productivity. It comprises different kinds of habitats including open areas with strong upwelling and complex water circulation presenting high dynamics in the upper layer, such as the Strait of Sicily [32], [34], as well as semi-closed basins with shallow waters such as the North Aegean Sea [1].

The habitat behaviour of small pelagic fish has been extensively studied mainly in the upwelling or highly productive ecosystems (e.g. European Atlantic waters or the Humboldt Current) where the presence of high abundance of anchovy and sardines at different life stages is often associated with high chlorophyll and zooplankton concentrations [e.g. 38] as well as specific ranges of salinity, temperature, sea level anomaly, temperature and oxygen stratifications and other oceanographic variables [e.g. 3,7,38,39]. But what about less productive ecosystems like the Mediterranean ? Are there any common behavioral pattern and which are the driving factors ? The current work aims to address these questions. A large environmental dataset, composed by *in situ* measurements and satellite data, has been used here to study the habitat selection behavior of anchovy and sardine in two areas within the Mediterranean Sea: the Strait of Sicily and the North Aegean Sea. Obtained results highlighted how different environmental regimes can result into a common habitat selection behavior for small pelagics.

During early summer sardine population in the Mediterranean Sea is a mixture of both juveniles (age 0) and adults (ages 1, 2 and older). Therefore, summer distribution grounds reflect both nurseries and feeding areas. In both study areas, sardine exhibits a stronger selective behavior, showing higher densities at shallower waters (up to 70 m depth in the Strait of Sicily and up to 50 m depth in the North Aegean Sea) whereas anchovy shows selective behavior up to the 100 m isobath. Sardine juveniles’ potential habitat in the Mediterranean is known to be narrow in extent, generally patchier, and mostly located at inshore waters [27]. Anyway, higher probability of finding adult sardine during summer is also identified in waters deeper than 65 m depth [4], indicating common bathymetric preferences for both juveniles and adults in accordance to our findings. This is not the case in the Bay of Biscay, where adults and juveniles of European sardine separate their niches. Adults distribute offshore over the continental shelf along the shelf break [40], whereas only young fish are found inshore.

In the Mediterranean during summer, anchovy stocks are dominated by spawning adults and spawning areas are known to be mainly driven by a depth gradient [1], [10], [11], [22], [34], [41], [42]. In particular, in the Strait of Sicily anchovy spawning grounds mostly occurred within the 100 m isobath [34], [43], while in the North Aegean Sea Somarakis and Nikolioudakis [22] identified two major spawning grounds: the first one located in the eastern area influenced by an anticyclone, the Samothraki gyre (SG), and the second one in the Thermaikos gulf in association to river mouths.

**Figure 9:**
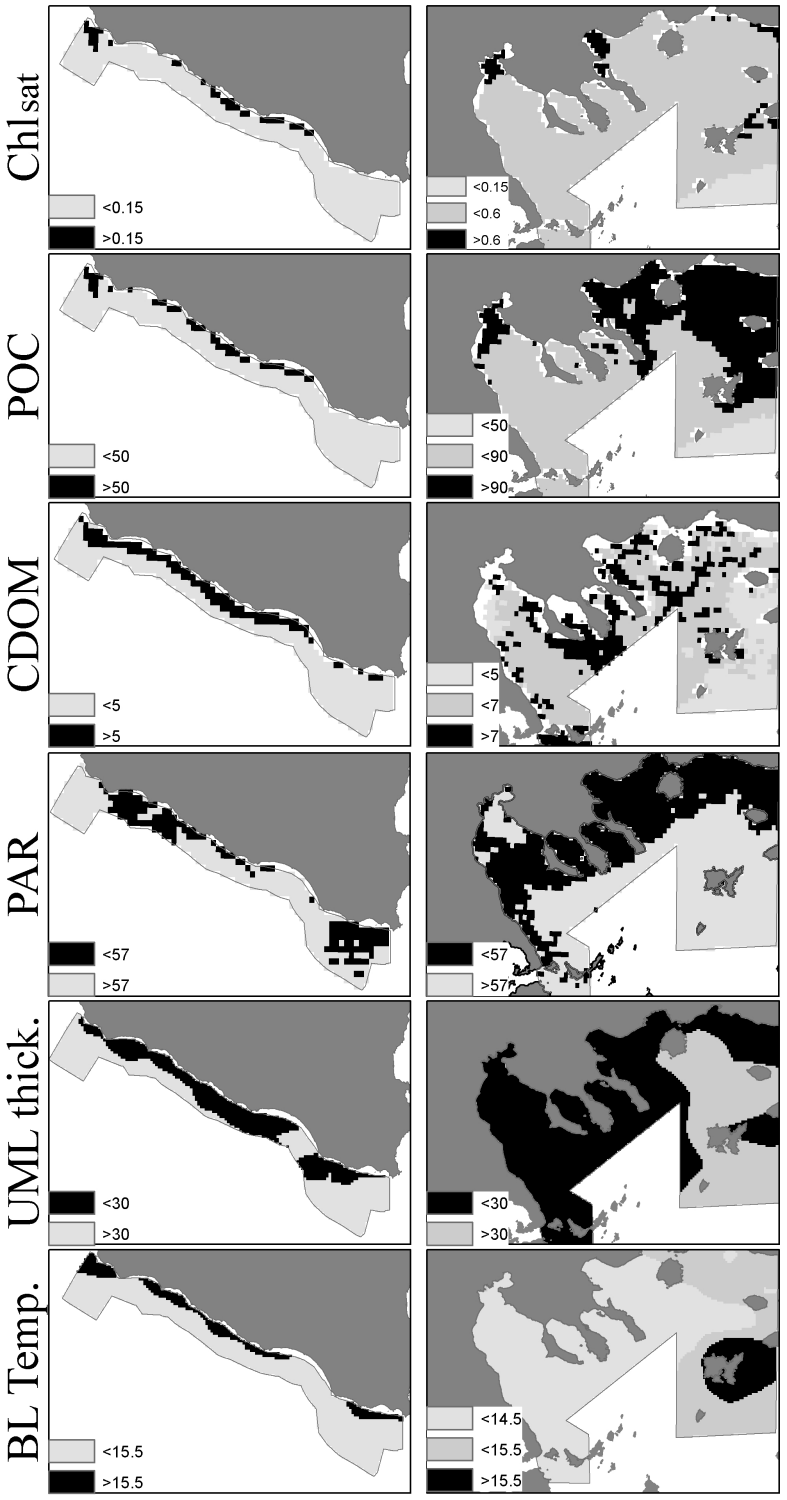
Distribution maps (2006 year) of Chl_sat_, POC, CDOM, PAR, UML thickness and BL temperature highlighting the differences in distribution patterns between the two areas. Black areas correspond to the selected ranges in each area.

Common selection patterns in the two study areas are observed in the case of BL temperature, UML thickness, POC, CDOM index and Chl_sat_. Specifically, both species prefer BL temperatures above 15.5°C and waters characterized by UML thickness up to 25 m, in both study areas. Less extended upper mixed layers are associated with the occurrence of upwelling processes, so this selection possibly reflects the preference for moderate upwelling in both areas. Selection for higher CDOM index, Chl_sat_ and POC concentrations is also pronounced for both species. This common trend is driven by the high demand for food in oligotrophic environments like the study areas, although the selected values in the Strait of Sicily (Chl_sat_>0.15 mg/m^3^ and POC >52 mg/m^3^) are smaller than the ones in the North Aegean Sea (Chl_sat_>0.4 mg/m^3^ and POC >130 mg/m^3^). This aspect is connected to the different ranges observed for these environmental variables in the two areas: lower productivity of the southern Sicilian waters [32] and higher Chl_sat_ and POC values recorded in the North Aegean Sea due to inputs of BSW, the extended continental shelf and rivers runoff [1], [22]. Although the range of values of the CDOM index is similar in both areas, CDOM largely differentiates in terms of its spatial pattern, with high CDOM values extending over a wide area in North Aegean Sea and limited to coastal waters in the Strait of Sicily. As an example, such differences between the two areas in terms of spatial extension of some investigated variables, highly affecting habitat suitability of both species, are shown in Fig. 9.

Whilst the QI analysis helped to highlight differences and similarities among areas and species in terms of selection/tolerance/avoidance for specific ranges of investigated variables, the PCA analysis results verified that the circulation regimes are largely responsible for the habitat selection patterns observed in both areas. In the Strait of Sicily the formation of a weak, coastal upwelling (represented into the lower PED, smaller values of UML thickness and shallow waters) is well associated with variables related to higher food availability (like CDOM, CHL_sat_, POC) explaining most of area variability. In the North Aegean Sea, analysis also verified the presence of two separate environmental regimes accounting for food availability in this area. First, the presence of BSW input, as reflected in low BL and UML salinity values, low UML thickness and higher CDOM, CHL_sat_, POC values. Secondly, the formation of a weak coastal upwelling, corresponding to shallow and less stratified waters (lower PED), is also related with higher CHL_sat_ and POC.

Furthermore, the two species differentiate their behavior concerning the effect of temperature in the upper water layer. In the North Aegean Sea both species prefer higher UML temperatures whereas the behavior is different in the Strait of Sicily where sardine seems to select warmer waters and anchovy colder ones. Moreover, in the Strait of Sicily, both species show an avoidance behavior for lower UML salinity values (<37.4) corresponding to the values characterizing the AIS core [44]. Concerning BL salinity, different behavior of both species is observed in the two areas. In the North Aegean Sea both species generally prefer salinity less than 38. In the Strait of Sicily anchovy selects a BL salinity range with values slightly higher than sardine, even though both species avoid higher values measured in such area. Sardine selects lower KE values, corresponding to more coastal waters (lower depths), and avoids KE values higher than 200 cm^2^s^−2^, indicating strong currents. A wider depth range selected by anchovy in the Strait of Sicily is associated also to KE range of intermediate values, showing at the same time a general tolerance behavior for a large range of current speeds. In the North Aegean Sea, it is only anchovy that clearly shows a selection behavior for lower UML salinity values, associated with water masses influenced by the BSW and/or rivers outflow. Species’ behavior in terms of PED also reflects local circulation conditions. Anchovy differentiates its behavior, selecting less stratified waters in the Strait of Sicily, possibly associated with moderate upwelling conditions, whereas selects well stratified waters in the North Aegean Sea. Sardine seems to select less stratified waters or otherwise moderate upwelling conditions in both study areas.

A clear difference between the two study areas, in terms of habitat selection, is singled out also by the quotient curves of MADT values. In particular, both species in the Strait of Sicily show an avoidance behavior for higher (>6 cm) MADT values corresponding to the area nearest the AIS path. Practically, although both species show a general preference for relatively shallower waters over the continental shelf, the AIS path acts like a physical barrier limiting their spatial distribution as both species tend to avoid higher current speeds and higher MADT. Often the AIS flows very close to the coast and leads to the formation of a dense front that entraps mesozooplankton concentrations, maintaining high food availability within coastal areas [34], [45]. MADT values in the Strait of Sicily are well correlated with UML thickness (r = 0.5; p<0.05), probably due to the effects of upwelling phenomena or movement of the Modified Atlantic Water causing frontal structures along the southern Sicilian coast. In the North Aegean Sea there is not a clear preference/avoidance behavior in terms of MADT. Instead, anchovy seems to select a range of values corresponding to moderate downwelling processes. Such values often characterize the peripheral part of areas in the margins of anti-cyclonic formations like the Samothraki gyre, typically found in Thracian plateau associated with BSW input [6], [22].

Concerning PAR, a common selection behavior for values less than 55 Einstein m^−2 ^day^−1^ was observed for both species at both study areas but it was found significant only in North Aegean Sea. In this region, the spatial distribution of this level of PAR values coincides with areas associated with high Chl_sat_ concentrations like river outflows and the location of the permanent anticyclone formed at the Thracian Sea plateau. PAR is indicative of the solar energy available for photosynthesis, controlling the growth of phytoplankton and thus critical also for fisheries and carbon dynamics. It is often used to determine the euphotic depth in the ocean (defined as the depth of 1% of the surface radiation), taking into account light attenuation and absorption. However, particularly high values of PAR are known to cause a significant decrease in cell abundance [46]. It is clear that the common trends in habitat selection of anchovy and sardine, observed in two ecosystems with marked differences in hydrodynamics, are driven by the oligotrophic character of the two regions. Practically, we identified a preference of both species for areas where shallow waters over the continental shelf meet suitable conditions for photosynthesis levels; such areas coincide with different circulation patterns that enhance productivity and subsequently food availability. Both anchovy and sardine select areas where the “ocean triad” hypothesis is generally met (i.e. enrichment, concentration and retention processes *sensu* Bakun [15]). Stratification, retention and plankton production have been proposed as controlling factors for anchovy spawning (or spawning success) in the Bay of Biscay [47]. Furthermore, the differences between the two species can be attributed to their life cycle and the sampling period targeting the optimization of spawning for anchovy and the selection of suitable juvenile grounds for sardine. Thus, sardine selects the coastal, shallower and warmer waters of both areas, in agreement with previous findings [4], [27], [48]. In the case of anchovy, our study highlighted a clear selection for areas presenting moderate upwelling or downwelling features like the periphery of anticyclones i.e. the Samothraki gyre in North Aegean Sea, the periphery of the AIS in the Strait of Sicily, enhancing frontal formation and retention for mesozooplankton, eggs and larvae [34], [42], [49].

Extensive research effort has been made to understand the factors determining anchovy and sardine habitat in upwelling areas where fish habitat expands or shrinks, largely determined by the intensification of the upwelling. In southern African waters anchovy spawns from midshelf to the offshore area of the shelf, within waters of intermediate temperature (17–21°C) and high salinity [9], [50], [51] clearly separating its niche from adult sardine [52]. In south Australia anchovies spawn in productive waters (Chl-α concentration up to 4.5 mg m^–3^) and temperatures ranging from 15.5 to 23.5°C but anchovies tend to expand their distribution to shelf waters only when sardine abundance is low [53], [54]. In the California Current, Northern anchovy spawns all year round and spawning usually is observed nearshore (up to 100 nmi from the coast), in waters with low temperatures (12–18°C), high salinity (33.5–33.75) and high primary production [55], [56]. The Peruvian anchovy or anchoveta (*Engraulis ringens*) distribution is generally restricted to cold coastal water masses characterized by high productivity and large plankton [57]. Anchovies habitat in the Humboldt System is strongly related to the strength and the duration of El Nino events in this area. The northern and central stocks spawn on the continental shelf (up to the 100 m isobath), over a wide temperature range (14–21°C), in areas characterized by intense upwelling and high primary production [58], [59]. Anchovies also spawn in the fjords of southern Chile (42° –47°S), areas characterized by highly stratified, stable waters that favor larval growth and survival [60].

Although the “ocean triad” hypothesis was initially observed in upwelling areas, it seems well applicable also to coastal areas where physical processes that increase productivity, are considered mostly responsible for the spatial organisation of plankton concentration and subsequently drive the spatial distribution patterns of anchovy and sardine [34], [45], [61]. However, the strength and the extent of such oceanographic regimes are adjusted to the peculiarities of each ecosystem. The main difference with other more productive ecosystems (e.g. southern Africa, California Current, Humboldt Current, Black Sea) is not related to differences in the preferential range for variables like temperature or salinity, but is mainly related to the absence of extended horizontal migrations for small pelagic fish in the Mediterranean. In this basin anchovies and sardines do not perform long migrations between feeding, spawning and juvenile grounds. These areas seem to overlap to a big extent, opposed to upwelling ecosystems. Moreover, the present study has shown that sardine and anchovy habitats show a large degree of overlap, in accordance to previous findings [1], [27]. The current work has shown that although the wide distribution area of the two species largely overlaps in the two ecosystems in question, it is largely driven by the local productivity patterns. Opposed to the upwelling ecosystems, habitat expansion is less pronounced between years of low and high abundance, since the suitable habitat even in years of high abundance remains within the rich food or the food entrapping areas.
